# Poly-L-Lactic Acid Attenuates Fibrotic Remodeling During Cutaneous Wound Healing

**DOI:** 10.3390/ijms27198874

**Published:** 2026-10-05

**Authors:** Kyung-A Byun, Hyoung Moon Kim, Seyeon Oh, Ki Woong Yu, Ji Min Lee, Kuk Hui Son, Kyunghee Byun

**Affiliations:** 1 Department of Anatomy & Cell Biology, College of Medicine, Gachon University, Incheon 21936, Republic of Korea; 2 Functional Cellular Networks Laboratory, Lee Gil Ya Cancer and Diabetes Institute, Gachon University, Incheon 21999, Republic of Korea; 3 LIBON Inc., Incheon 22006, Republic of Korea; 4 Maylin Apgujeong Clinic, Seoul 06024, Republic of Korea; 5 Maylin Clinic Jamsil Lotte Hotel Branch, Seoul 05554, Republic of Korea; 6 Director Global Clinic Gangnam, Seoul 06611, Republic of Korea; 7 Department of Thoracic and Cardiovascular Surgery, Gachon University Gil Medical Center, Gachon University, Incheon 21565, Republic of Korea; 8 Department of Health Sciences and Technology, Gachon Advanced Institute for Health & Sciences and Technology (GAIHST), Gachon University, Incheon 21999, Republic of Korea

**Keywords:** Collagen remodeling, Epithelial–mesenchymal transition, Hypoxia, Poly-L-lactic acid

## Abstract

Pathological hypoxia following cutaneous injury is a critical driver of wound chronicity and fibrotic remodeling. The nuclear receptor binding SET domain protein 2 (NSD2)/lysine demethylase 2A (KDM2A)–histone H3 lysine 36 dimethylation (H3K36me2) axis is a known regulator of hypoxia-induced epithelial-to-mesenchymal transition (EMT) in malignancy; however, its role in skin repair remains largely unexplored. We investigated whether poly-L-lactic acid (PLLA) could attenuate fibrotic remodeling by modulating the NSD2/KDM2A–H3K36me2 axis and examining angiogenic signaling. We used a murine 9 mm punch-wound model for in vivo assessment and HaCaT keratinocytes and human dermal fibroblasts (HDFs) exposed to 2% O_2_ as in vitro hypoxia models. PLLA was administered to evaluate its impact on angiogenic signaling, collagen architecture, and the NSD2/KDM2A pathway. PLLA was associated with increased angiogenic markers and reduced expression of several hypoxia-associated epigenetic and fibrotic markers. In mice, PLLA increased vascular endothelial growth factor (VEGF)/VEGF receptor 2 expression and blood vessel abundance. In addition, PLLA decreased NSD2 and H3K36me2 levels while increasing KDM2A levels. Histological analysis showed less dense collagen staining and a change in the collagen type I/type III staining ratio, alongside a smaller photographically defined residual lesion area. Under controlled hypoxic conditions in vitro, PLLA suppressed EMT-related transcription factors, including Snail, Slug, and Twist, in keratinocytes and attenuated transforming growth factor-β1/phosphorylated mothers against decapentaplegic homolog 2/3 signaling and α-smooth muscle actin expression in fibroblasts. These findings support further study of PLLA-associated changes in vascular, epithelial, and fibrotic markers during wound remodeling.

## 1. Introduction

Cutaneous wound healing is a highly coordinated process that proceeds via overlapping inflammatory, proliferative, and remodeling phases. In addition to macroscopic wound closure, proper cutaneous wound healing requires granulation tissue formation, collagen maturation, scar resolution, and re-epithelialization [1,2]. Disturbance of the tightly regulated interaction among keratinocytes, fibroblasts, endothelial cells, immune cells, and extracellular matrix during wound repair can redirect healing toward wound chronicity or fibrosis [3]. Oxygen supply is a central determinant of this process, because it supports cell survival, angiogenesis, growth-factor signaling, collagen biosynthesis, matrix cross-linking, and, ultimately, restoration of tissue function [1].

Development of a transient hypoxic gradient is a normal physiological response following injury, as the oxygen demand from heightened metabolic activity surpasses the delivery capacity of disrupted blood vessels [1]. When vascular recovery is inadequate, this early adaptive state transitions into persistent pathologic hypoxia, which impairs wound closure, prolongs inflammation, disturbs matrix turnover, and predisposes the tissue to fibrosis or wound chronicity [1,4]. Hypoxia is particularly relevant to fibroblast biology because it can amplify transforming growth factor-beta 1 (TGF-β1)/mothers against decapentaplegic homolog (Smad) signaling and enhance collagen production in dermal fibroblasts, thereby promoting a synthetic or myofibroblast-like program that is closely linked to excessive scar formation [4]. In parallel, type 2 epithelial–mesenchymal transition (EMT) contributes to wound healing but must remain transient and spatially restricted to avoid maladaptive remodeling [5]. When EMT-associated transcription factors such as Snail, Slug, and Twist are inappropriately activated, epithelial identity is weakened, mesenchymal traits become reinforced, and the wound microenvironment shifts toward persistent fibrotic remodeling rather than orderly regeneration [5].

Recent evidence suggests that hypoxia stabilizes the state transitions required for proper wound healing through epigenetic mechanisms as well as canonical cytokine signaling [6,7]. Of particular interest is the epigenetic pathway involving the nuclear receptor binding SET domain protein 2 (NSD2)/lysine demethylase 2A (KDM2A)–histone H3 lysine 36 dimethylation (H3K36me2) axis. NSD2 functions as the primary writer of H3K36me2, while KDM2A serves as its eraser. Fluctuations in H3K36me2 govern the progression of epithelial cells along the epithelial-to-mesenchymal spectrum [8]. Although the NSD2/KDM2A–H3K36me2 axis is a well-established driver of hypoxic EMT in malignancy [7,8,9], its potential role in cutaneous repair remains largely unexplored, representing a significant gap in our understanding of wound chronicity.

Given that cancer metastasis and pathological wound healing share fundamental signaling pathways governing cellular plasticity, findings from malignancy models provide a plausible framework for cutaneous repair. In pancreatic ductal adenocarcinoma models, loss of NSD2 reinforces epithelial identity, whereas KDM2A deficiency promotes a mesenchymal phenotype, establishing H3K36me2 as a global epigenomic determinant of plasticity rather than simply a bystander [8]. Furthermore, hypoxia has been shown to drive sustained EMT by suppressing KDM2A and stabilizing NSD2 through a histone-methylation–mitogen-activated protein kinase (MAPK) signaling loop, thereby reinforcing H3K36me2-dependent mesenchymal reprogramming [6]. Recently, hypoxic pancreatic fibroblasts were shown to accumulate H3K36me2 and use a nuclear factor κ-light-chain-enhancer of activated B cells (NF-κB)/H3K36me2 program to enhance secretion of EMT-supportive growth factors, suggesting that the NSD2/KDM2A–H3K36me2 axis can affect both the epithelial and the stromal compartments [9]. 

The literature regarding the use of biomaterials to promote wound healing has largely focused on whether biomaterials improve wound closure, angiogenesis, inflammation, epithelial coverage, collagen deposition, or gross scar appearance, rather than whether they interrupt hypoxia-driven chromatin remodeling [10,11,12,13,14]. Poly(lactic acid) (PLA)-based biomaterials are commonly evaluated on the basis of wound area repair, collagen deposition, angiogenesis, and cell activity [10]. Representative studies of poly(L-lactic acid) (PLLA) and poly(D,L-lactic acid) (PDLLA) have emphasized piezoelectric or structural support effects, pro-angiogenic activity, re-epithelialization, Yes-associated protein (YAP) inhibition, collagen synthesis, oxidative-stress control, macrophage modulation, and scar remodeling outcomes [11,12,13,14]. We hypothesized that PLLA improves wound healing through a dual mechanism: by altering angiogenic markers and hypoxia-associated pathways, and by attenuating hypoxia-driven fibroblast synthetic conversion and keratinocyte EMT through regulation of TGF-β/Smad signaling and the NSD2/KDM2A–H3K36me2 axis. To test this hypothesis, we combined PLLA dose-optimization experiments with a murine 9 mm punch-wound model and mechanistic studies using keratinocyte and fibroblast 2% O_2_ hypoxia models.

## 2. Results

### 2.1. PLLA Enhances Angiogenic Signaling in Wound Tissue

**Figure 1:**
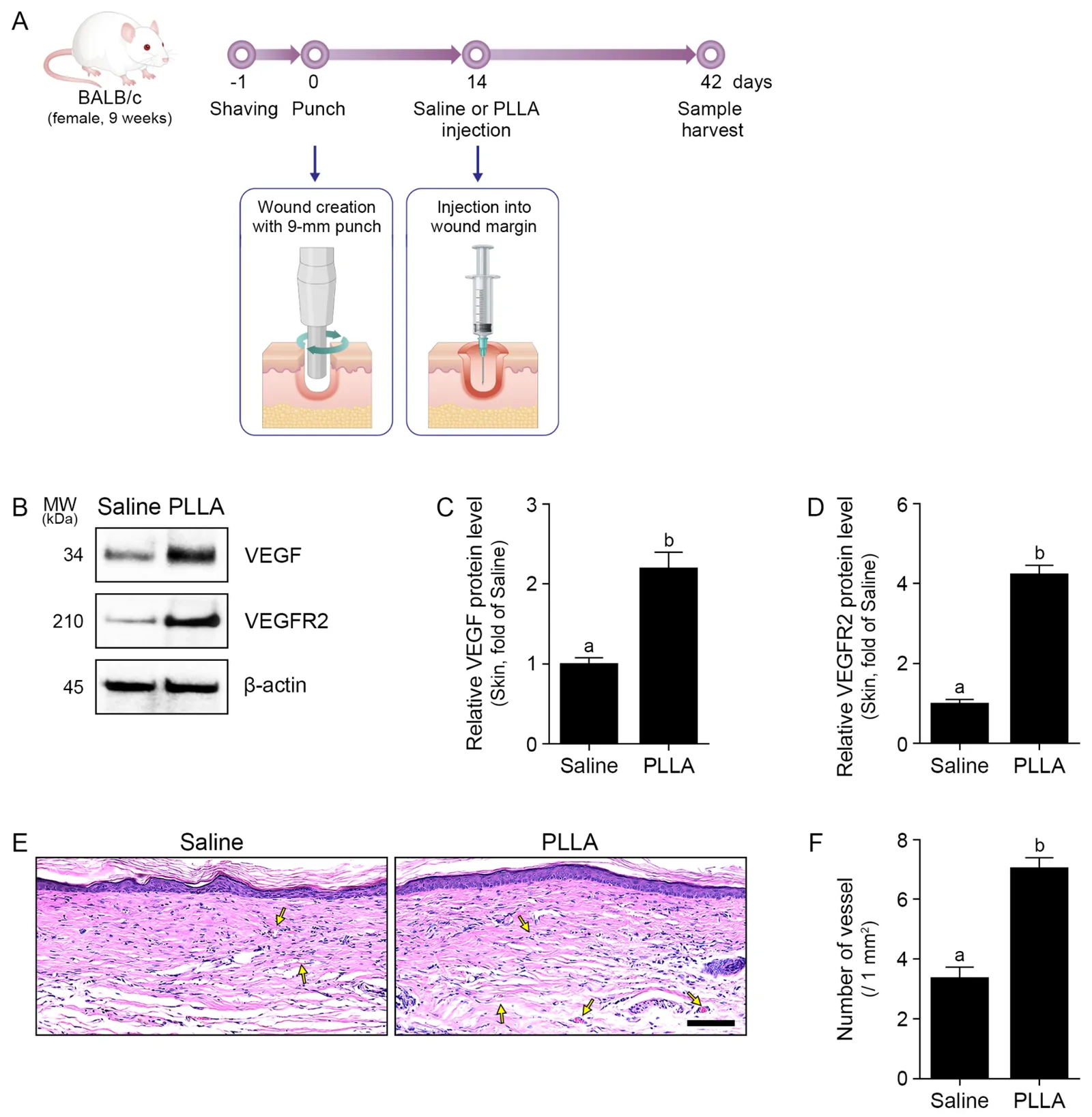
PLLA promotes angiogenesis in wounded skin tissue. (**A**) Schematic illustration of the in vivo wound-healing model. A full-thickness excisional wound was generated on the dorsal skin of mice. Fourteen days after wound induction, mice received intradermal injections of saline or PLLA (50 mg in 6 mL saline; 100 μL/cm^2^). Skin tissues were harvested 28 days after treatment. (**B**) Western blot showing VEGF and VEGFR2 in wound tissues following PLLA treatment. β-actin was used as a loading control. (**C**,**D**) Quantitative analysis of the VEGF (**C**) and VEGFR2 (**D**) protein expression shown in (**B**). (**E**) Representative H&E-stained images of wound tissues. Scale bar = 100 μm. Arrows indicate blood vessels. (**F**) Quantification of vessel number in wound tissues based on H&E staining. Data are presented as mean ± SD (*n* = 5 mice per group). Statistical significance was assessed using the Mann–Whitney U test. Different letters indicate statistically significant differences between groups (*p* < 0.05). H&E, hematoxylin and eosin; MW, molecular weight; PLLA, poly-L-lactic acid; SD, standard deviation; VEGF, vascular endothelial growth factor; VEGFR2, vascular endothelial growth factor receptor 2.

To determine whether PLLA alters the vascular response during wound remodeling, we established a murine wound model using a 9 mm punch injury followed by local administration of saline or PLLA (50 mg in 6 mL saline), with tissue collection at day 28 after PLLA administration (Figure 1A). In our murine model, PLLA significantly increased the expression of VEGF and vascular endothelial growth factor receptor 2 (VEGFR2) compared with the saline control (Figure 1B–D). Histological analysis with hematoxylin and eosin (H&E) staining confirmed a higher number of vessel-like structures in PLLA-treated tissues compared with control tissues (Figure 1E,F).

### 2.2. PLLA Regulates the NSD2/KDM2A–H3K36me2 Epigenetic Axis and Attenuates Fibrotic Signaling in Wound Tissue

**Figure 2:**
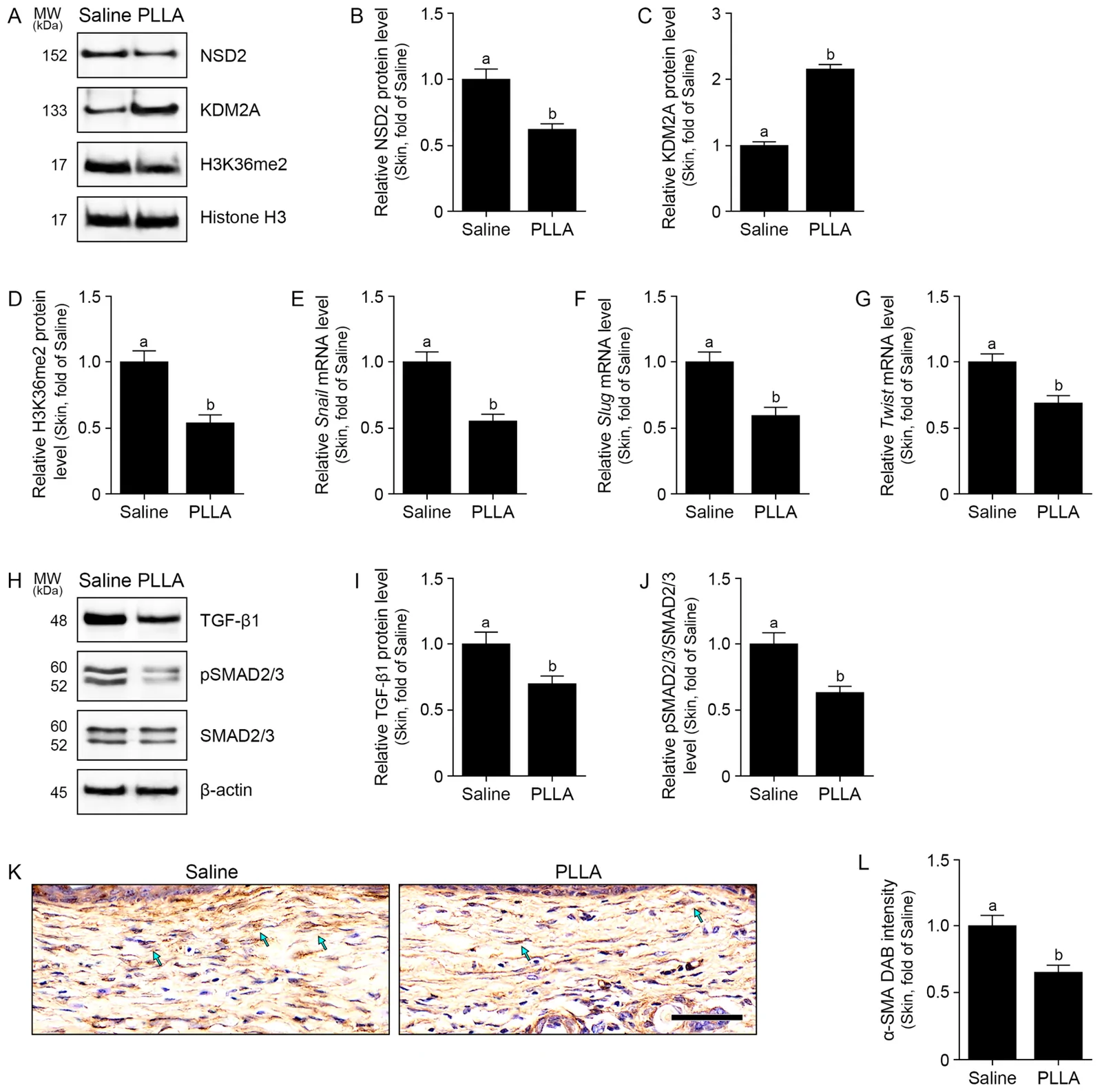
PLLA regulates EMT-associated signaling and fibroblast synthetic phenotype in wounded skin tissue. (**A**) Representative Western blot images showing nuclear NSD2, KDM2A, and H3K36me2 expression levels in wound tissues following PLLA treatment. Histone H3 was used as a nuclear protein loading control. (**B**–**D**) Quantitative analysis of the Western blot results for NSD2 (**B**), KDM2A (**C**), and H3K36me2 (**D**) expression shown in (**A**). (**E**–**G**) Quantitative analysis of Snail (**E**), Slug (**F**), and Twist (**G**) mRNA expression. (**H**) Representative Western blot images showing TGF-β1, pSMAD2/3, and SMAD2/3 expression levels in wound tissues following PLLA treatment. β-actin was used as a loading control. (**I**,**J**) Quantitative analysis of the Western blot results for TGF-β1 (**I**) and pSMAD2/3/SMAD2/3 (**J**) expression is shown in (**H**). (**K**) Representative immunohistochemistry images of α-SMA expression in wound tissues. Scale bar = 100 μm. (**L**) Quantification of the α-SMA DAB intensity shown in (**K**). Data are presented as mean ± SD (*n* = 5 mice per group). Statistical significance was assessed using the Mann–Whitney U test. Different letters indicate statistically significant differences between groups (*p* < 0.05). α-SMA, α-smooth muscle actin; DAB, 3,3′-diaminobenzidine; EMT, epithelial-to-mesenchymal transition; H3K36me2, histone H3 lysine 36 dimethylation; KDM2A, lysine demethylase 2A; mRNA, messenger RNA; MW, molecular weight; NSD2, nuclear receptor-binding SET domain protein 2; PLLA, poly-L-lactic acid; pSMAD2/3, phosphorylated SMAD2/3; SD, standard deviation; SMAD2/3, mothers against decapentaplegic homolog 2/3; TGF-β1, transforming growth factor-β1.

We next investigated whether PLLA influences the epigenetic landscape associated with wound chronicity. Western blot analysis of nuclear fractions from wound tissues demonstrated that PLLA treatment decreased NSD2 expression and H3K36me2 while concurrently increasing KDM2A levels (Figure 2A–D). Additionally, PLLA treatment downregulated EMT-related transcription factors, including Snail, Slug, and Twist (Figure 2E–G) and suppressed the TGF-β1/phosphorylated SMAD2/3 (pSMAD2/3) signaling pathway (Figure 2H–J). The expression of α-smooth muscle actin (α-SMA), a marker of myofibroblast differentiation, was also significantly reduced in the PLLA group (Figure 2K,L).

### 2.3. PLLA Attenuated EMT-Associated Signaling in Hypoxic Keratinocytes and Fibrotic Markers in Hypoxic Fibroblasts

To establish an optimal in vitro model for wound hypoxia, we exposed HaCaT keratinocytes and human dermal fibroblasts (HDFs) to 2% O_2_ over a time course (Figure S1A). In HaCaT cells, we observed a time-dependent increase in NSD2 expression coupled with a decrease in KDM2A levels (Figure S1B,C). In HDFs, hypoxia induced a progressive increase in TGF-β1 expression (Figure S1D). Notably, these specific markers in both cell types showed statistically significant changes starting from 12 h of hypoxia exposure. Based on these findings, PLLA was administered 12 h after the start of hypoxia in subsequent experiments.

We performed dose–response experiments to confirm the optimal concentration of PLLA (Figure S2A). Cell-viability assays indicated no significant cytotoxicity in either HaCaT cells or HDFs exposed to up to 1 mg/mL PLLA under hypoxic conditions (Figure S2B,C). Analysis of mRNA expression showed that a PLLA concentration of 0.4 mg/mL most effectively normalized NSD2 and KDM2A levels in keratinocytes and suppressed TGF-β1 induction in HDFs, so this concentration was selected for subsequent mechanistic studies (Figure S2D–F).

**Figure 3:**
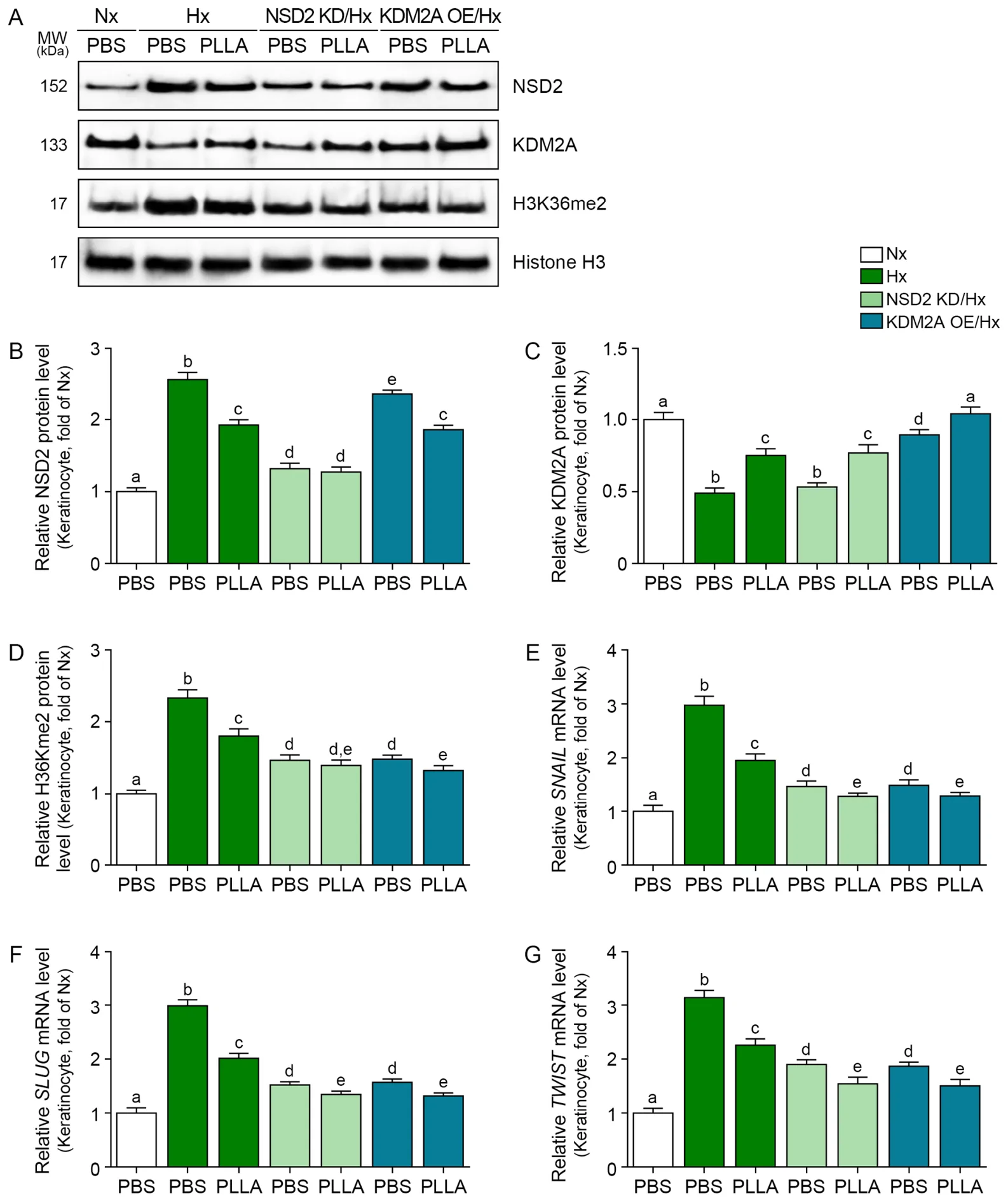
PLLA regulates EMT-associated signaling under hypoxic conditions in keratinocytes. (**A**) Western blot images showing nuclear NSD2, KDM2A, and H3K36me2 expression levels in HaCaT cells. Histone H3 was used as a nuclear protein loading control. (**B**–**D**) Quantitative analysis of the Western blot results for NSD2 (**B**), KDM2A (**C**), and H3K36me2 (**D**) expression shown in (**A**). (**E**–**G**) Quantitative analysis of *SNAIL* (**E**), *SLUG* (**F**), and *TWIST* (**G**) mRNA expression. Data are presented as mean ± SD (*n* = 5 independent experiments). Statistical differences were assessed using the Kruskal–Wallis test, followed by pairwise Mann–Whitney U tests. Different letters indicate statistically significant differences between groups (*p* < 0.05). EMT, epithelial-to-mesenchymal transition; H3K36me2, histone H3 lysine 36 dimethylation; Hx, hypoxia; KD, knockdown; KDM2A, lysine demethylase 2A; mRNA, messenger RNA; MW, molecular weight; NSD2, nuclear receptor-binding SET domain protein 2; Nx, normoxia; OE, overexpression; PBS, phosphate-buffered saline; PLLA, poly-L-lactic acid; SD, standard deviation.

**Figure 4:**
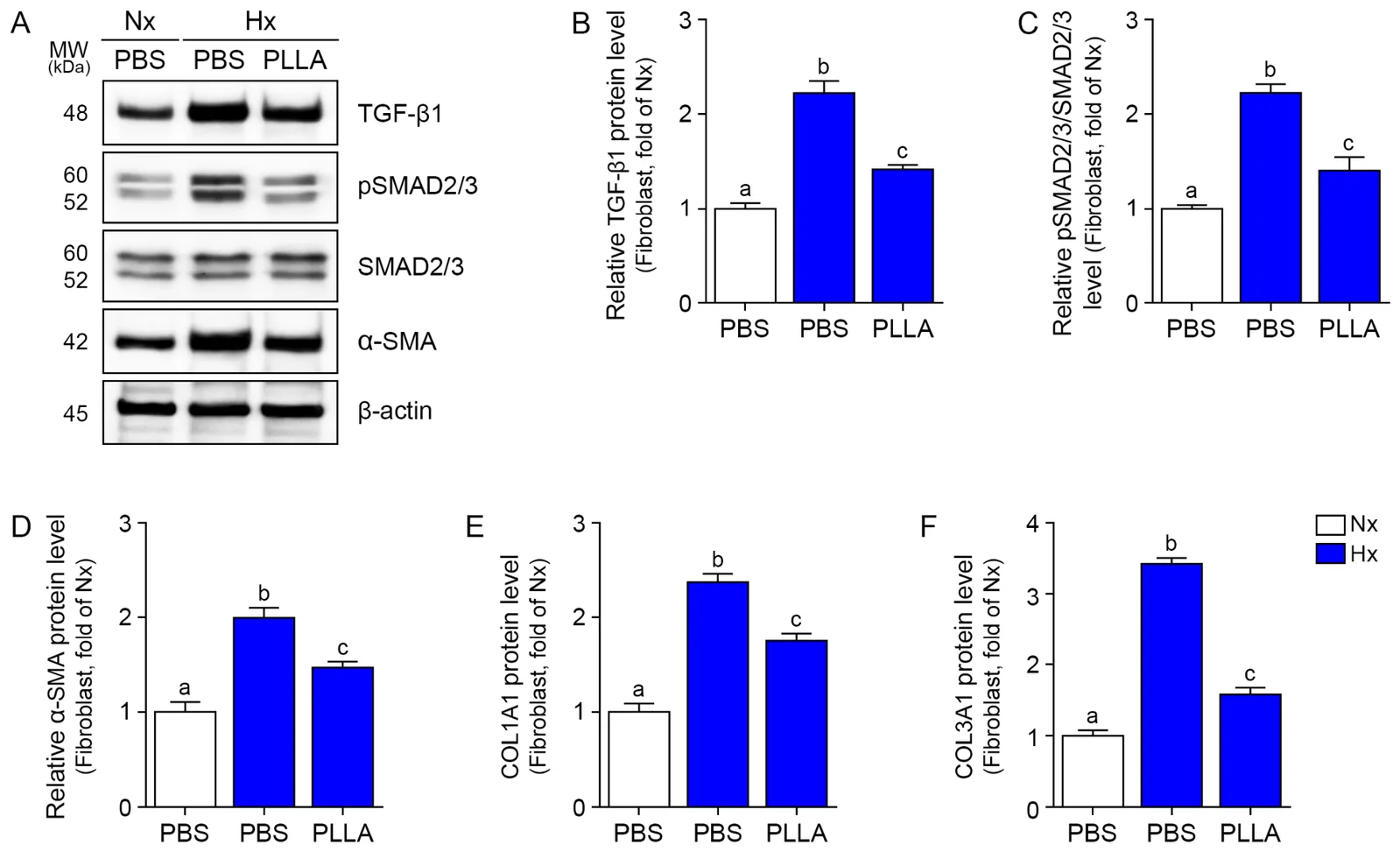
PLLA regulates the synthetic phenotype of fibroblasts under hypoxic conditions. (**A**) Western blot images showing TGF-β1, pSMAD2/3, SMAD2/3, and α-SMA expression levels in HDFs. β-actin was used as a loading control. (**B**–**D**) Quantitative analysis of the Western blot results for TGF-β1 (**B**), pSMAD2/3/SMAD2/3 (**C**), and α-SMA (**D**) expression shown in (**A**). (**E**,**F**) Relative protein levels of collagen type I (**E**) and type III (**F**) in HDF cell lysate, measured by ELISA. Data are presented as mean ± SD (*n* = 5 independent experiments). Statistical differences were assessed using the Kruskal–Wallis test, followed by pairwise Mann–Whitney U tests. Different letters indicate statistically significant differences between groups (*p* < 0.05). α-SMA, α-smooth muscle actin; ELISA, enzyme-linked immunosorbent assay; HDFs, human dermal fibroblasts; Hx, hypoxia; MW, molecular weight; Nx, normoxia; PBS, phosphate-buffered saline; PLLA, poly-L-lactic acid; pSMAD2/3, phosphorylated SMAD2/3; SD, standard deviation; SMAD2/3, mothers against decapentaplegic homolog 2/3; TGF-β1, transforming growth factor-β1.

Using the conditions established in the dose–response experiments, we examined corresponding signaling changes in vitro. In hypoxic HaCaT cells, PLLA treatment effectively reversed the hypoxia-induced upregulation of NSD2 and downregulation of KDM2A, in parallel with decreased H3K36me2 levels and lower expression of Snail, Slug, and Twist, with similar directional changes observed after NSD2 knockdown or KDM2A overexpression (Figure 3 and Figure S3). In HDFs, PLLA treatment attenuated hypoxia-induced activation of the TGF-β1/pSMAD2/3 pathway and reduced α-SMA expression (Figure 4A–D and Figure S4). Furthermore, ELISA results indicated that PLLA significantly reduced the protein levels of collagen type I and type III in lysates of hypoxic HDFs (Figure 4E,F).

### 2.4. PLLA Alters Collagen Staining and Reduces Residual Lesion Area

**Figure 5:**
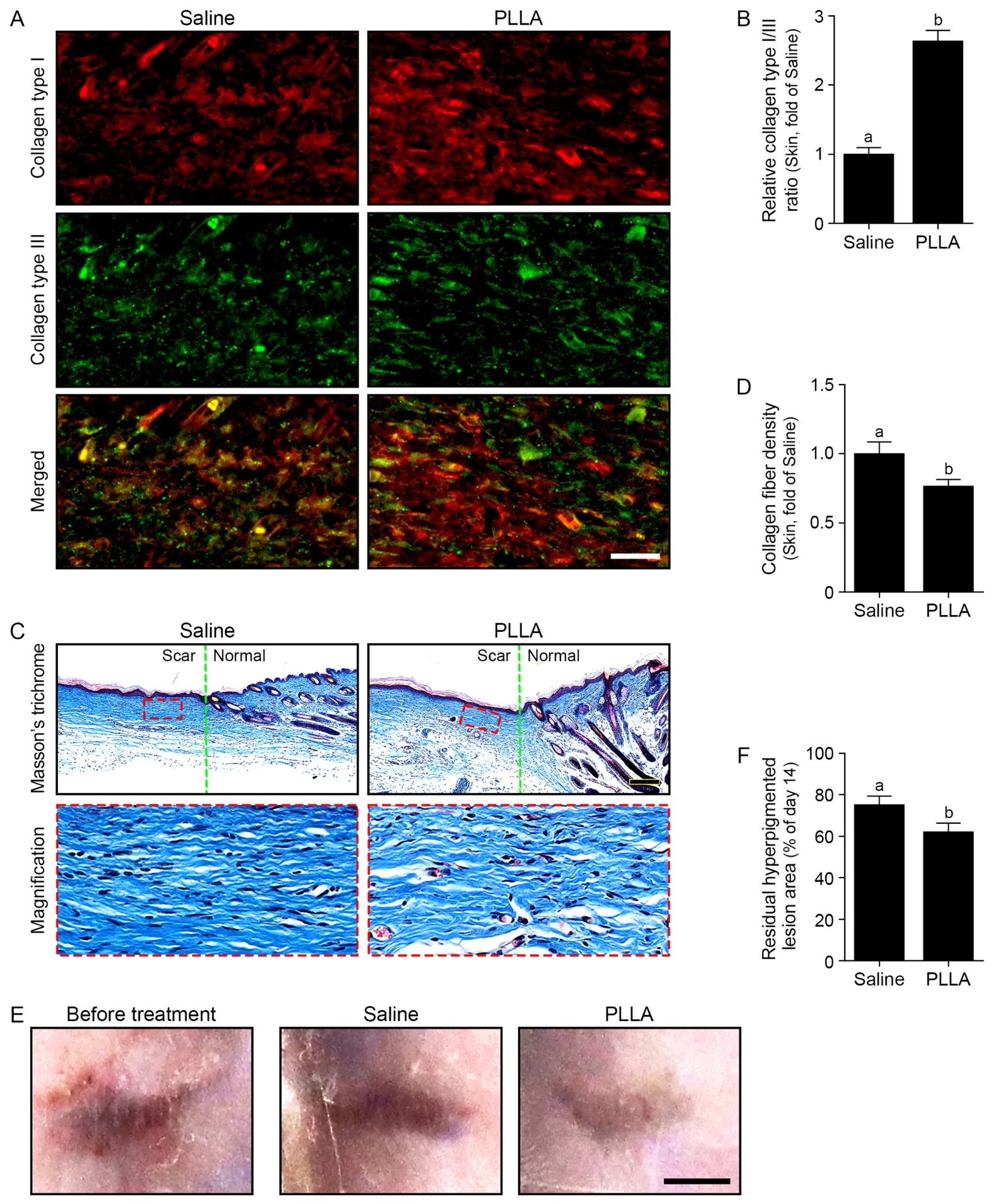
PLLA alters collagen staining and reduces residual hyperpigmented lesion area in wounded skin. (A) Representative immunofluorescence images showing collagen type I (red) and type III (green) expression in wound tissues following PLLA treatment. Merged images are shown in the lower panels. Scale bar = 25 μm. (**B**) For each mouse, collagen I-positive area was divided by collagen III-positive area in 50 μm × 50 μm regions of interest in (**A**); the resulting mouse-level ratios were normalized to the saline group. (**C**) Representative Masson’s trichrome (MT) staining images of wound tissues. Collagen fibers are stained blue. Dashed boxes indicate magnified regions. Scale bar = 200 μm. (**D**) Quantification of collagen density in wound tissues based on MT staining. (**E**) Representative gross images showing the lesion before treatment at day 14 and following saline or PLLA treatment at day 42. The pretreatment panel illustrates the appearance of the residual lesion; quantitative ratios were calculated using each mouse’s own day-14 lesion area. Scale bar = 2 mm. (**F**) Visible hyperpigmented lesion-area ratio calculated for each mouse as (day-42 lesion area/day-14 lesion area) × 100 from photographs in (**E**); both time points follow initial wound closure. An example of the photographic lesion boundary is shown in Figure S5. Data are presented as mean ± SD (*n* = 5 mice per group). Statistical significance was assessed using the Mann–Whitney U test. Different letters indicate statistically significant differences between groups (*p* < 0.05). MT, Masson’s trichrome; PLLA, poly-L-lactic acid; SD, standard deviation.

We next examined collagen staining and the visible residual lesion. Pretreatment day-14 hyperpigmented lesion areas did not differ significantly between the saline and PLLA groups (Mann–Whitney U test, *p* = 0.84). Wounds treated with saline exhibited disordered and densely packed collagen fibers, whereas PLLA-treated wounds appeared less densely stained, with a more interlaced appearance in the representative images (Figure 5A). Quantitative analysis revealed that PLLA changed the mouse-level collagen type I/type III positive-area ratio after normalization to the saline group (Figure 5B), decreased the collagen density (Figure 5C,D), and reduced the day-42/day-14 photographically defined hyperpigmented lesion-area ratio (Figure 5E,F). These findings are consistent with altered matrix remodeling after PLLA treatment; collagen fiber organization and skin function were not directly quantified.

## 3. Discussion

Our study is consistent with changes in the wound microenvironment at the vascular, fibroblastic, and epithelial levels. In the murine model, PLLA increased VEGF/VEGFR2 expression and vessel abundance. In hypoxic fibroblasts and keratinocytes, PLLA attenuated profibrotic and EMT-associated signaling pathways. Together, these observations suggest related vascular, epithelial, and fibroblast responses, without establishing their causal order. This integrated interpretation is physiologically significant because wound quality is ultimately determined not simply by the rate of surface closure but by the ability of the tissue to successfully exit the reactive repair program and enter a stable remodeling phase characterized by restored structure and function [15].

A major conceptual focus of our study is the emphasis on the NSD2/KDM2A–H3K36me2 axis as a candidate pathway. While the biological significance of NSD2, KDM2A, and H3K36me2 is well established in the context of cancer-related epithelial plasticity, metastatic progression, and hypoxic cancer-associated fibroblast signaling [7,8,9], this epigenetic axis is rarely considered as an organizing mechanism of cutaneous repair in wound healing. This focus on cutaneous wound healing distinguishes our findings from prior reports on PLA and PLLA biomaterials. Rather than limiting our scope to downstream outcomes such as macroscopic closure rates or physical scaffold support, we attempted to elucidate how a biomaterial can influence markers associated with hypoxic chromatin remodeling and cell-state transitions [10,11,12,13,14,16]. The keratinocyte data, in particular, provide an important mechanistic layer to our study. In hypoxic keratinocytes, PLLA reduced nuclear NSD2 and H3K36me2 levels while restoring KDM2A expression, a shift that was directly accompanied by reductions in the EMT-related transcription factors Snail, Slug, and Twist. The inclusion of keratinocytes with NSD2 knockdown or KDM2A overexpression in our experiments supports the involvement of the NSD2/KDM2A axis. Our results suggest that rather than merely reducing EMT markers as a secondary effect of improved healing, PLLA-associated changes involve this pathway in hypoxic keratinocytes; its necessity for the PLLA response remains untested.

The mechanistic plausibility of our hypothesis is further strengthened by existing evidence regarding hypoxia-induced NSD2 stabilization and KDM2A inhibition. Prior research demonstrated that hypoxia directly impairs the catalytic activity of the KDM2A demethylase and suppresses the expression of the protein phosphatase 2 (PP2)-family phosphatases responsible for NSD2 degradation [6]. Src family kinase (SFK)/MAPK signaling activation under hypoxia further stabilizes NSD2, reinforcing an H3K36me2-dependent transcriptional landscape [6]. Collectively, our data suggest that PLLA treatment is associated with changes in the balance between the epigenetic writer (NSD2) and eraser (KDM2A), alongside lower H3K36me2 under the tested hypoxic conditions.

Whether PLLA changes oxygenation in vivo remains unknown; the observed H3K36me2 and EMT-marker changes are consistent with, but do not establish, the proposed pathway. Our current data align with this framework; however, future investigations should more directly characterize the biochemical turnover of NSD2. Specifically, further studies utilizing cycloheximide-chase assays to determine protein half-life, or ubiquitin-proteasome inhibition assays to confirm degradation pathways, would be beneficial [6,17]. Additionally, exploring the role of PP2Cδ-mediated dephosphorylation or utilizing phospho-mutant NSD2 constructs may clarify the precise biochemical events underlying the PLLA-associated chromatin changes.

Our findings have further implications concerning collagen remodeling. Collagen deposition alone is not a sufficient sign of good healing, because a wound can close and still mature into a functionally inferior scar if the extracellular matrix remains excessive, poorly organized, or mechanically abnormal [3]. During normal repair, the early provisional matrix is relatively enriched in type III collagen, but successful remodeling gradually restores a more collagen type I-dominant dermis [18]. Reported absolute values vary by analytic method, anatomic site, and age, but the collagen content in adult dermis is generally described as being roughly 80–85% type I collagen and 8–11% type III collagen, whereas the scar-focused literature often cites a type I to type III collagen ratio of 4–5:1 in normal adult skin [19,20]. Hypertrophic scars have been reported to show a higher type I:III ratio, commonly around 6:1, whereas keloids can show even higher ratios, such as 17:1 [21]. These published composition estimates provide context but cannot define a normal reference for the present fluorescence ratio, which lacked matched unwounded skin [3].

The collagen architecture is equally important as the types of collagen in the dermis, because normal dermis exhibits a reticular or basket-weave organization, whereas pathologic scars show denser, thicker, more parallel, and less physiologically interlaced collagen bundles [22]. Accordingly, when we describe improved collagen remodeling in this study, the most defensible interpretation is that the representative sections appear less densely stained; fiber alignment and basket-weave architecture require dedicated quantitative analysis.

Wound quality also depends on how completely the skin architecture is re-established. In murine histologic scoring systems, late or complete remodeling is associated with restoration of dermal white adipose tissue, reappearance of multiple skin appendages, and regeneration of panniculus carnosus muscle within the wound area [15]. Thus, in a preclinical full-thickness model, the re-emergence of adnexal or dermal structures suggests that healing has progressed beyond mere closure and toward tissue restoration [15]. Severe, full-thickness wounds in human adults usually heal with scar formation rather than true appendage regeneration, and scars typically lack normal hair follicles, sweat glands, and rete ridges [23,24]. Therefore, in the human context, appendage regeneration should be framed as an indicator of higher-quality regenerative healing rather than as an absolute minimum criterion for clinically acceptable closure [25]. Our findings show differences in selected remodeling markers, while appendage regeneration and restoration of normal skin architecture were not established.

The clinical importance of proper remodeling is underscored by the consequences of abnormal scar endpoints [26]. Mature scars can be classified as normotrophic, atrophic, or hypertrophic, and these different endpoints reflect distinct failures in matrix production, organization, and resolution [26]. Normotrophic scars are relatively flat and mature with less erythema over time, whereas atrophic scars are depressed because of insufficient fibrosis, and hypertrophic scars are raised, erythematous, firm, and typically remain confined within the original wound margin [27,28]. Hypertrophic scars may cause pruritus, pain, stiffness, contracture, reduced range of motion, cosmetic deformity, and psychosocial distress, especially when they occur across joints or on exposed sites such as the face [28,29]. These outcomes explain why reductions in myofibroblast persistence, tension-driven matrix alignment, and collagen overaccumulation should be viewed as clinically meaningful beyond basic wound closure [28].

Our murine model nevertheless has some important limitations. The dorsal punch-wound model does not naturally reproduce human hypertrophic scarring, because mouse skin is relatively loose and contains the panniculus carnosus, which drives rapid wound contraction and makes closure less dependent on the prolonged granulation-tissue and re-epithelialization dynamics that dominate human healing [30]. As a result, unsplinted murine wounds tend to underrepresent tension-dependent scar maturation and rarely generate spontaneous hypertrophic scars that closely resemble the human condition [31]. Despite this, punch and excisional murine wound models are inexpensive, surgically simple, reproducible, compatible with genetically modified mice, and highly suitable for time-course molecular analysis and first-pass therapeutic screening [32]. Within that framework, our 9 mm punch generated a full-thickness excisional wound that permitted evaluation of remodeling-associated markers; published murine excisional-wound studies support the reproducibility of this model class and the utility of histological analysis, although they do not specifically validate the 9 mm diameter [33,34]. Nevertheless, increasing the wound size alone does not abolish contraction bias, and human-like hypertrophic scar modeling is more directly approached with splinted wounds or tension-enforced systems such as suture-anchored models [35]. Therefore, the fairest interpretation is that our 9 mm punch model does not solve the species gap, and is useful for examining remodeling-associated biology in vivo.

There are additional limitations to our study. First, the cell experiments used an acute 2% O_2_ model that does not fully capture the spatiotemporal oxygen gradients, inflammatory mediators, and biomechanical heterogeneity of a living wound bed. Second, the strongest mechanistic literature on NSD2/KDM2A–H3K36me2 comes from studies of epithelial plasticity and hypoxic EMT in the context of cancer rather than classical cutaneous wound-healing models, which means that direct chromatin-level validation in primary skin cells remains an important next step. Third, because no direct wound oxygenation measurement, matched unwounded collagen reference, quantitative fiber-orientation analysis, or rescue/opposing perturbation was included, the vascular, epigenetic, and matrix findings cannot establish a single causal sequence. Fourth, the NSD2 knockdown and KDM2A overexpression experiments also lacked matched negative transfection controls, limiting attribution of the observed changes to the intended constructs. Fifth, animal allocation was not randomized, and image and laboratory outcome analyses were not blinded, leaving potential allocation and observer bias. Because collagen remodeling involves composition, alignment, cross-linking, and mechanics, future work would be strengthened by adding Picrosirius red or second-harmonic generation imaging, tensile testing, and formal scar-elevation or remodeling scoring rather than relying on collagen markers alone.

Nevertheless, our data support a model in which PLLA improves wound healing by altering angiogenic, keratinocyte, fibroblast, and matrix-associated markers. Our findings motivate further tests of the contribution of the NSD2/KDM2A–H3K36me2 axis to the PLLA response.

## 4. Materials and Methods

### 4.1. Preparation of PLLA Material

PLLA microparticles (McCoom7; SACCI Bio Co., Ltd., Seoul, Republic of Korea) were supplied by the manufacturer and sterilized by electron-beam irradiation before use. Manufacturer-provided molecular weight, particle morphology, particle-size distribution, and morphological stability data are presented in Figure S6. Detailed characterization procedures and manufacturer-reported cytotoxicity information are described in Supplementary Methods.

### 4.2. In Vivo Experiments

All animal procedures were conducted in accordance with institutional guidelines and were approved by the Institutional Animal Care and Use Committee of Gachon University (IACUC; LCDI-2025-0042). This study complied with the ethical standards of the Association for Assessment and Accreditation of Laboratory Animal Care International (AAALAC International, Frederick, MD, USA) and adhered to the Animal Research: Reporting of In Vivo Experiments (ARRIVE) guidelines. Each mouse was injected and analyzed individually throughout the study. No humane endpoints were reached during the study.

Female BALB/c mice (8 weeks old) were purchased from Orient Bio (Seongnam, Republic of Korea) and acclimatized for one week before the experiment. Mice were housed under controlled environmental conditions (temperature 22 ± 2 °C, humidity 50–60%) with a 12 h light/dark cycle and free access to food and water. To establish a wound-healing model, a full-thickness excisional wound (9 mm diameter) was created on the dorsal skin of anesthetized mice. Fourteen days after wound induction, when the wounds had closed, animals were assigned sequentially, without a random-number procedure, to the saline and PLLA groups (five mice per group). All assigned animals received the planned injection and were included in the final analysis; no animals were excluded. Mice received intradermal injections of either saline or PLLA (50 mg in 6 mL saline) at a dose of 100 μL/cm^2^ at the wound site. Skin tissues were collected 28 days after treatment for histological and molecular analyses.

The photographic analysis focused on the lesions remaining within the closed wounds rather than the rate of initial wound closure. Areas of hyperpigmented lesions—defined as regions darker in color than the surrounding skin—were marked on photographs taken at days 14 and 42 using ImageJ software (version 1.53s; National Institutes of Health, Bethesda, MD, USA). For each mouse, the relative lesion area at day 42 was calculated as (area of hyperpigmented lesion at day 42/area of hyperpigmented lesion at day 14) × 100. Thus, this metric represents a photographic measurement of the residual hyperpigmented lesion area rather than a histological measurement of the scar area [36].

### 4.3. In Vitro Experiments

Human HaCaT keratinocytes (RRID: CVCL_0038; kindly provided by Prof. Jeong-Hee Hong, Gachon University, Incheon, Republic of Korea) were cultured in Dulbecco’s modified Eagle medium (Gibco) containing 10% fetal bovine serum and 1% penicillin–streptomycin. Human dermal fibroblasts (HDFs; LOT: TWJ28-163, CEFO Co., Ltd., Seoul, Republic of Korea) derived from a 14-year-old male donor were cultured in CEFOgro^TM^ Human MSC Growth Medium (CEFO Co., Ltd.). HDFs at passages 4–7 were used. According to the manufacturer’s certificate of analysis, the HDFs expressed CD29 (98.7%) and CD90 (99.0%) and tested negative for mycoplasma contamination. These results represent manufacturer-provided cell characterization and mycoplasma testing. All cells were maintained in incubators at 37 °C with 5% CO_2_. To establish a hypoxia model, cells were cultured in a hypoxia incubator (2% O_2_) for the indicated time periods (6–60 h). Based on preliminary experiments, 12 h hypoxia exposures were selected for experiments because they induced consistent changes in target molecules (Figure S1). PLLA was suspended in phosphate-buffered saline (PBS) and diluted in serum-free medium to the indicated concentrations. For cytotoxicity assessment, cells were treated with PLLA at concentrations ranging from 0 to 10 mg/mL using the CCK-8 assay. For the CCK-8 assay, cell-free blank wells containing the corresponding PLLA concentrations and CCK-8 reagent were processed under the same conditions as the experimental wells. Absorbance was measured at 450 nm, and the absorbance of the corresponding concentration-matched blank was subtracted from each experimental value. For functional assays, cells were treated with 0–0.8 mg/mL PLLA (Figure S2). Based on preliminary dose–response experiments showing comparable effects above 0.4 mg/mL, a concentration of 0.4 mg/mL was used for subsequent experiments. For mechanistic studies in HaCaT keratinocytes, NSD2 knockdown and KDM2A overexpression were achieved by Lipofectamine-mediated transfection with NSD2-targeting shRNA (Santa Cruz Biotechnology, Inc., Dallas, TX, USA) and a KDM2A overexpression plasmid (Addgene, Watertown, MA, USA), respectively, prior to hypoxia exposure. Cells were harvested 48 h after PLLA treatment for subsequent analyses. HaCaT and HDF experiments were each performed on five independent occasions; PCR and ELISA samples were run in three wells per occasion. Matched negative transfection controls were not included for the NSD2 knockdown and KDM2A overexpression experiments.

### 4.4. Western Blot

Total and nuclear proteins were extracted using RIPA lysis buffer (ATTO Corporation, Tokyo, Japan) containing protease and phosphatase inhibitor cocktails and a nuclear extraction kit (Invitrogen, Carlsbad, CA, USA), respectively. Protein concentrations were measured using a bicinchoninic acid assay kit (Thermo Fisher Scientific). Equal amounts of protein (20–40 μg) were separated by sodium dodecyl sulfate–polyacrylamide gel electrophoresis and transferred onto polyvinylidene fluoride membranes (Millipore, Burlington, MA, USA), and incubated with primary antibodies (Table S1) and horseradish peroxidase (HRP)-conjugated secondary antibodies (Vector Laboratories, Burlingame, CA, USA). Protein bands were detected using an enhanced chemiluminescence detection system (Cytiva) and visualized using a ChemiDoc imaging system (Bio-Rad, Hercules, CA, USA). Band intensities were quantified using ImageJ software (NIH) and normalized to β-actin or histone H3 for total or nuclear proteins, respectively.

### 4.5. Histological Analysis

Skin tissue samples collected from mice were fixed in 4% paraformaldehyde for 72 h at 4 °C and embedded in paraffin. Paraffin blocks were sectioned at a thickness of 7 μm using a microtome and mounted on slides. The skin tissues were deparaffinized in xylene and rehydrated through a graded ethanol series (100%, 95%, 80%, 70%), followed by rinsing in distilled water. For H&E staining, sections were stained with hematoxylin (KPNT, Cheongju, Republic of Korea) and counterstained with eosin (KPNT). For Masson’s trichrome staining, collagen fibers were stained according to standard protocols to assess collagen deposition and fibrosis (KPNT). Images were acquired using a VS200 Slide Scanner (Olympus Corporation, Tokyo, Japan). Five wound-region fields per mouse were analyzed; adjacent unwounded skin was not used. Field measurements were averaged within each mouse before group comparisons [37]. Positive staining areas were quantified using ImageJ software (NIH) [38].

### 4.6. Immunohistochemistry

Paraffin-embedded skin sections were deparaffinized, rehydrated, and incubated with primary antibodies (Table S1). For immunofluorescence, sections were incubated with Alexa Fluor-conjugated secondary antibodies (Thermo Fisher Scientific, Waltham, MA, USA), and mounted with antifade mounting medium. For 3,3′-diaminobenzidine (DAB) staining, antigen retrieval was performed in citrate buffer (pH 6.0), followed by incubation with biotinylated secondary antibodies and VECTASTAIN Elite ABC reagent (Vector Laboratories, Burlingame, CA, USA). Immunoreactivity was visualized using DAB (Sigma-Aldrich, St. Louis, MO, USA) and counterstained with hematoxylin (KPNT). Images were acquired using a VS200 Slide Scanner (Olympus Corporation, Tokyo, Japan). Five wound-region fields per mouse were analyzed; adjacent unwounded skin was not used. Field measurements were averaged within each mouse before group comparisons [37]. Positive staining areas were quantified using ImageJ software (NIH).

For collagen I and III immunofluorescence, images were converted to RGB stacks, and the red and green channels corresponding to collagen I and III, respectively, were analyzed separately in ImageJ (NIH). A 50 μm × 50 μm region of interest (ROI) at the same location was applied to both channels within each wound-region field, excluding adjacent unwounded skin. Automatic thresholding was applied to each channel, and the percentage of the ROI occupied by threshold-positive pixels (%Area) was measured. The collagen I/collagen III positive-area ratio was calculated for each field by dividing the collagen I %Area by the collagen III %Area. Ratios from five fields were averaged to obtain one value per mouse, which was subsequently normalized to the mean mouse-level ratio of the saline group.

### 4.7. Quantitative Real-Time PCR

Total RNA was extracted from cells and skin tissues using RNAiso reagent (Takara, Kusatsu, Japan) according to the manufacturer’s instructions. RNA concentration and purity were determined using a spectrophotometer (NanoDrop; Thermo Fisher Scientific, Wilmington, DE, USA). Complementary DNA was synthesized from 1 μg total RNA using a reverse transcription kit (Takara) according to the manufacturer’s protocol. Quantitative real-time PCR (qPCR) was performed using SYBR Green PCR Master Mix (Takara) on a real-time PCR system (Applied Biosystems, Foster City, CA, USA). The amplification conditions consisted of an initial denaturation at 95 °C for 10 min, followed by 40 cycles of denaturation at 95 °C for 15 s and annealing/extension at 60 °C for 1 min. All genes (Table S2) were normalized to ACTB or Actb expression using the 2^−ΔΔCt^ method.

### 4.8. Enzyme-Linked Immunosorbent Assay

Equal amounts of total protein from HDF cell lysates were coated onto 96-well plates in 0.05 M carbonate-bicarbonate buffer and incubated overnight at 4 °C. The plates were washed three times with PBS containing 0.1% Tween-20 (PBST). To block nonspecific binding sites, plates were incubated with blocking buffer consisting of 1% bovine serum albumin in PBS for 1 h at room temperature. After blocking, primary antibodies (Table S1) were added to each well, and the plates were incubated for 6 h at room temperature. The plates were then washed, and HRP-conjugated secondary antibodies were added and incubated for 2 h. Color development was performed using tetramethylbenzidine substrate solution (Sigma-Aldrich, St. Louis, MO, USA), and the reaction was terminated with 2 N sulfuric acid. Absorbance was measured at 450 nm using a microplate reader. The results were expressed as fold changes relative to the control group.

### 4.9. Statistical Analysis

Sample size was determined based on previous studies using comparable experimental models and practical considerations. For tissue imaging analyses, five fields were averaged to obtain a single value for each mouse (*n* = 5 mice per group); individual fields were not treated as independent biological replicates. Cell experiments were performed on five independent occasions, with three technical replicate wells for qPCR and ELISA; the technical replicates were averaged to obtain a single value for each independent experiment (*n* = 5 independent experiments). Allocation was sequential rather than randomized, and analyses were performed with group identity known to the analyst. Statistical analyses were performed using SPSS version 26 (IBM Corp., Armonk, NY, USA). Nonparametric tests were used because of the small sample size (*n* = 5) [39,40]. Overall differences were assessed using the Kruskal–Wallis test, followed by the Mann–Whitney U test for pairwise comparisons. Data are presented as mean ± SD.

## 5. Conclusions

PLLA treatment was associated with changes in vascular, epithelial, fibroblast, and matrix-associated markers during cutaneous wound remodeling. PLLA enhanced VEGF/VEGFR2-associated angiogenic responses and modulated the NSD2/KDM2A–H3K36me2 axis, changes that were associated with attenuated EMT and fibrotic signaling. The accompanying change in collagen staining and smaller photographically defined residual lesions are consistent with altered remodeling. Direct measurement of oxygenation and pathway rescue experiments are needed to test the proposed mechanism.

## Data Availability

The original contributions presented in this study are included in the article/Supplementary Materials. Further inquiries can be directed to the corresponding author(s).

## Supplementary Materials

The following supporting information can be downloaded at https://www.mdpi.com/article/10.3390/ijms27198874/s1.
