# Supplementary material for: Poly-L-Lactic Acid Attenuates Fibrotic Remodeling During Cutaneous Wound Healing
Source: Int J Mol Sci. 2026 Oct 5;27(19):8874. doi: 10.3390/ijms27198874 (PMC13665362; doi:10.3390/ijms27198874)
Supplement: Supplementary file 1 [file ijms-27-08874-s001.zip › ijms-4568278-supplementary.pdf]

Supplementary Figures

z

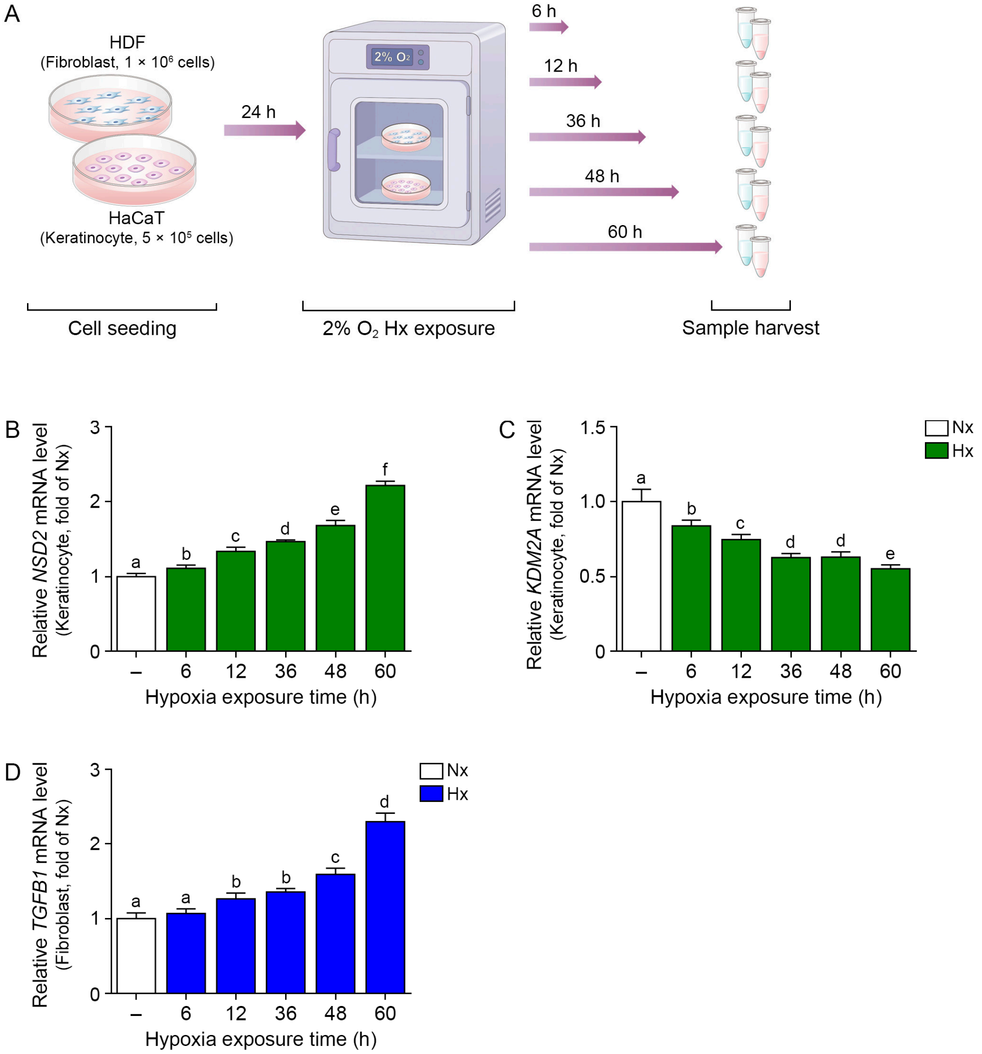

Figure S1. Establishment of hypoxic conditions in keratinocytes and fibroblasts. (A) Schematic illustration of the experimental design used to establish hypoxic conditions in HaCaT keratinocytes and HDFs. Cells were exposed to hypoxia (2% O_2) for 6, 12, 36, 48, or 60 h, followed by sample collection at each time point. (B,C) Quantitative analysis of *NSD2* (B) and *KDM2A* (C) mRNA expression in HaCaT keratinocytes. (D) Quantitative analysis of *TGFB1* mRNA expression in HDFs. Data are presented as mean ± SD (n = 5 independent experiments). Statistical differences were assessed using the Kruskal–Wallis test, followed by pairwise Mann–Whitney U tests. Different letters indicate statistically significant differences between groups ($p < 0.05$). HDFs, human dermal fibroblasts; Hx, hypoxia; KDM2A, lysine demethylase 2A; mRNA, messenger RNA; NSD2, nuclear receptor-binding SET domain protein 2; Nx, normoxia; SD, standard deviation; TGFB1, transforming growth factor-β1.

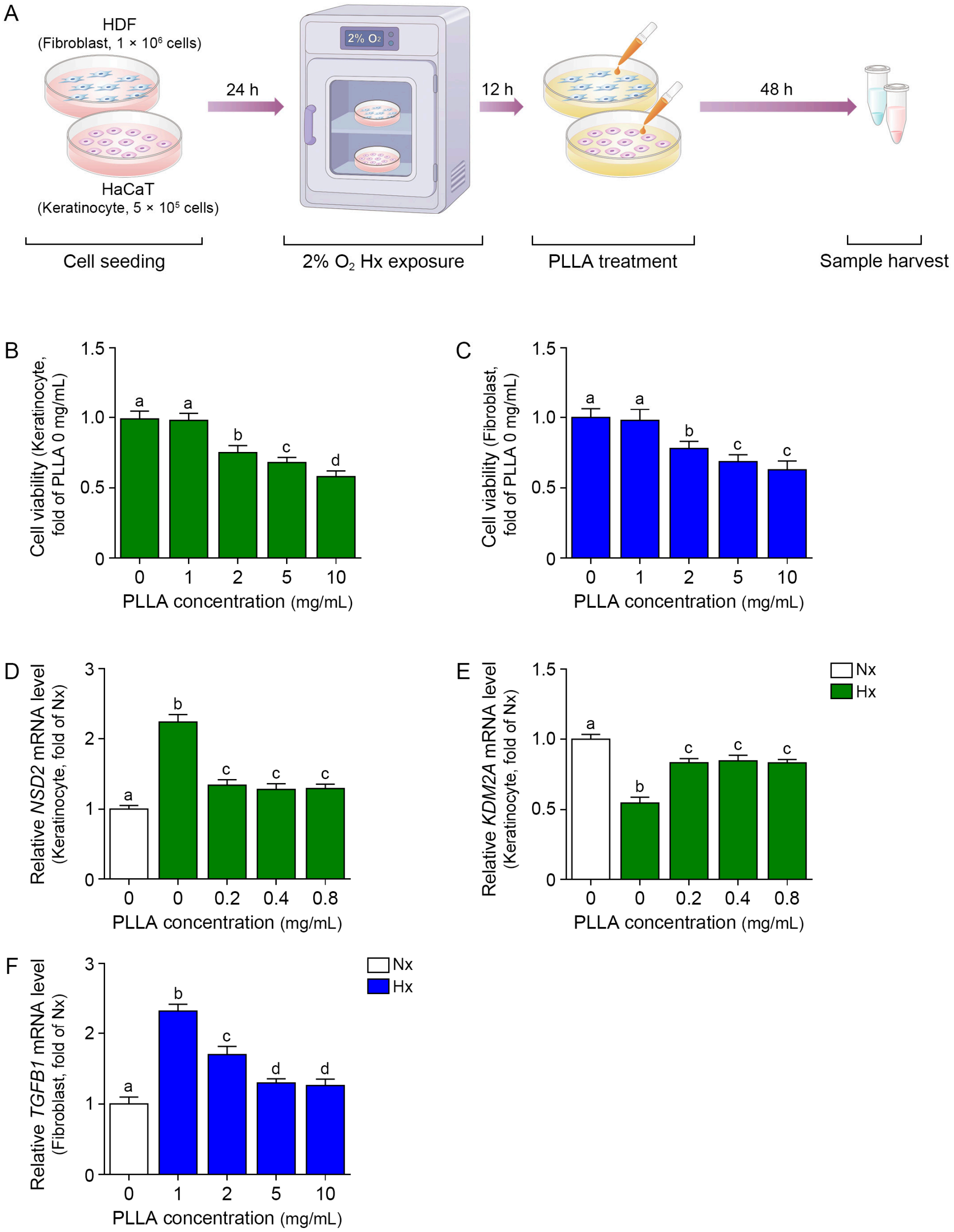

Figure S2. Determination of the optimal PLLA concentration for keratinocytes and fibroblasts under hypoxic conditions. (A) Schematic illustration of experimental design. HaCaT keratinocytes and HDFs were exposed to hypoxia (2% O_2) for 12 h, followed by PLLA treatment for 48 h. (B,C) Viability of HaCaT keratinocytes (B) and HDFs (C) treated with PLLA at the indicated concentrations. (D,E) Quantitative analysis of *NSD2* (D) and *KDM2A* (E) mRNA expression in HaCaT keratinocytes treated with PLLA. (F) Quantitative analysis of *TGFB1* mRNA expression in HDFs treated with PLLA at the indicated concentrations. Data are presented as mean ± SD (n = 5 independent experiments). Statistical differences were assessed using the Kruskal–Wallis test, followed by pairwise Mann–Whitney U tests. Different letters indicate statistically significant differences between groups ($p < 0.05$). HDFs, human dermal fibroblasts; Hx, hypoxia; KDM2A, lysine demethylase 2A; mRNA, messenger RNA; NSD2, nuclear receptor-binding SET domain protein 2; Nx, normoxia; PLLA, poly-L-lactic acid; SD, standard deviation; TGF-β1, transforming growth factor-β1.

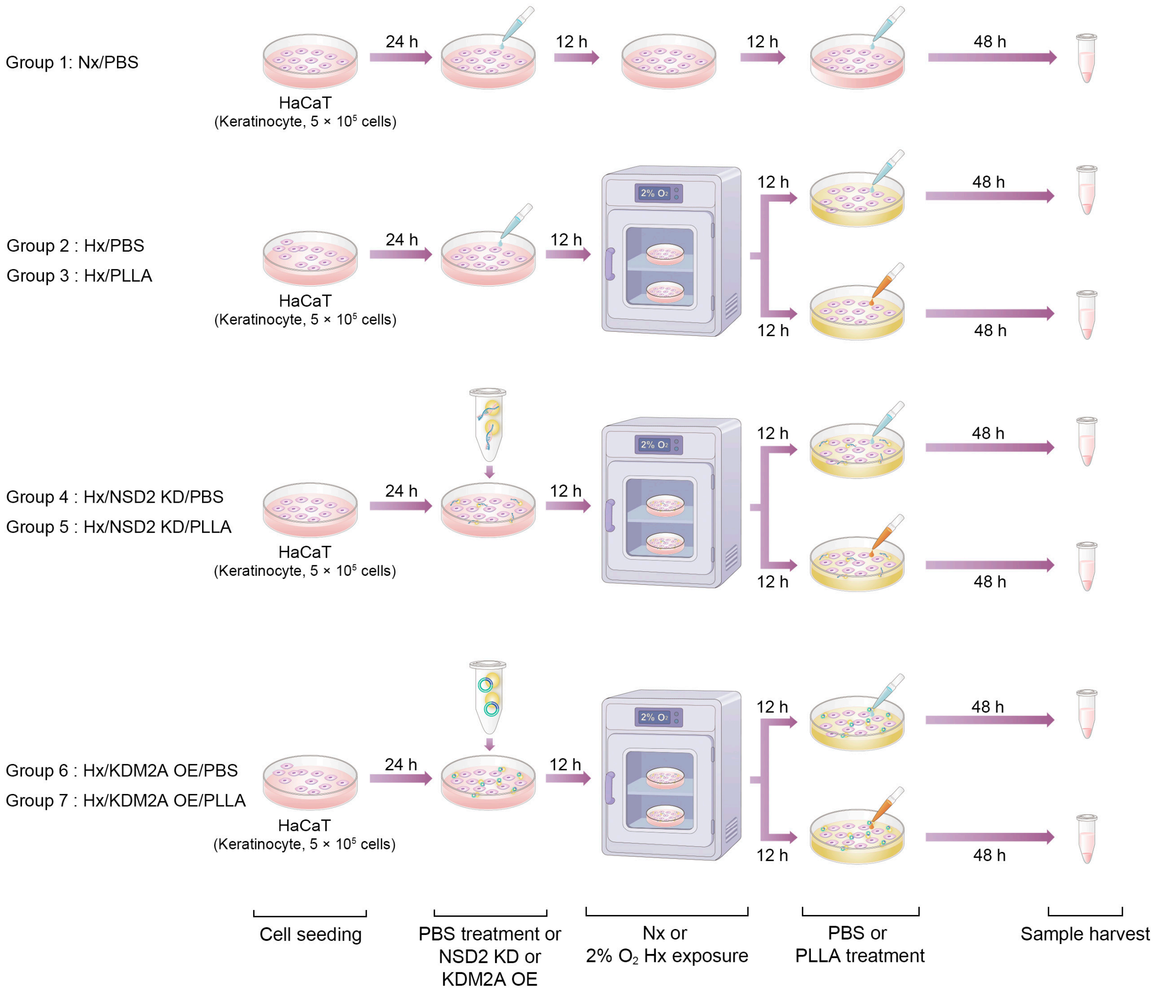

Figure S3. Schematic illustration of the experimental design for HaCaT keratinocytes. Cells were cultured under normoxic (21% O_2) or hypoxic (2% O_2) conditions and treated with PLLA (0.4 mg/mL). For mechanistic studies, NSD2 knockdown or KDM2A overexpression was performed prior to hypoxia exposure. Hx, hypoxia; KD, knockdown; KDM2A, lysine demethylase 2A; NSD2, nuclear receptor-binding SET domain protein 2; Nx, normoxia; OE, overexpression; PBS, phosphate-buffered saline; PLLA, poly-L-lactic acid.

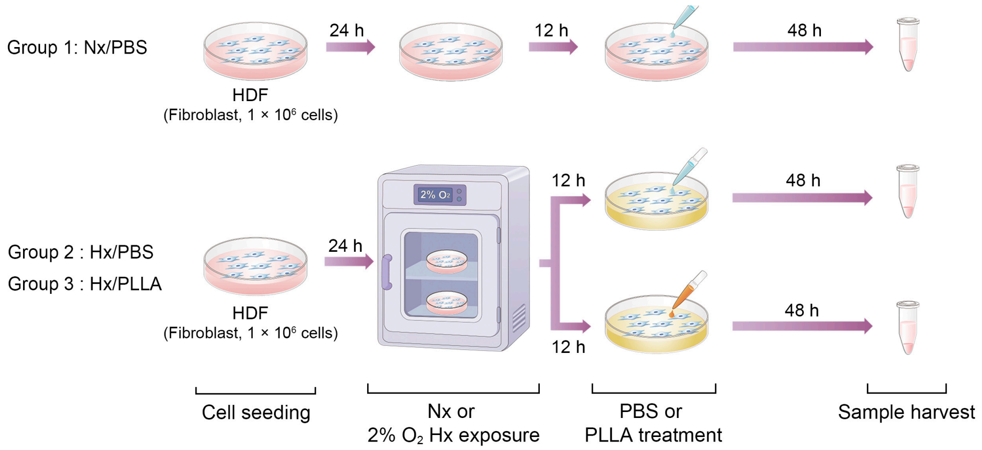

Figure S4. Schematic illustration of the experimental design for HDFs cultured under normoxic or hypoxic conditions with PLLA treatment. HDFs, human dermal fibroblasts; Hx, hypoxia; Nx, normoxia; PBS, phosphate-buffered saline; PLLA, poly-L-lactic acid.

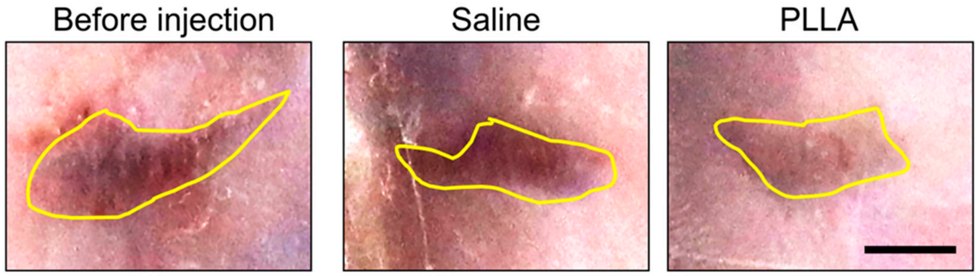

Figure S5. Representative photographic delineation of residual hyperpigmented lesions. Yellow outlines indicate lesion boundaries, defined by visibly darker coloration than the surrounding skin. The panels illustrate the boundary method and are not a separate quantitative baseline comparison. "Before injection" shows day 14 after wound creation, when the wound had closed; saline and PLLA panels show day 42, corresponding to 28 days after injection. Scale bar as shown in the original image. PLLA, poly-L-lactic acid.

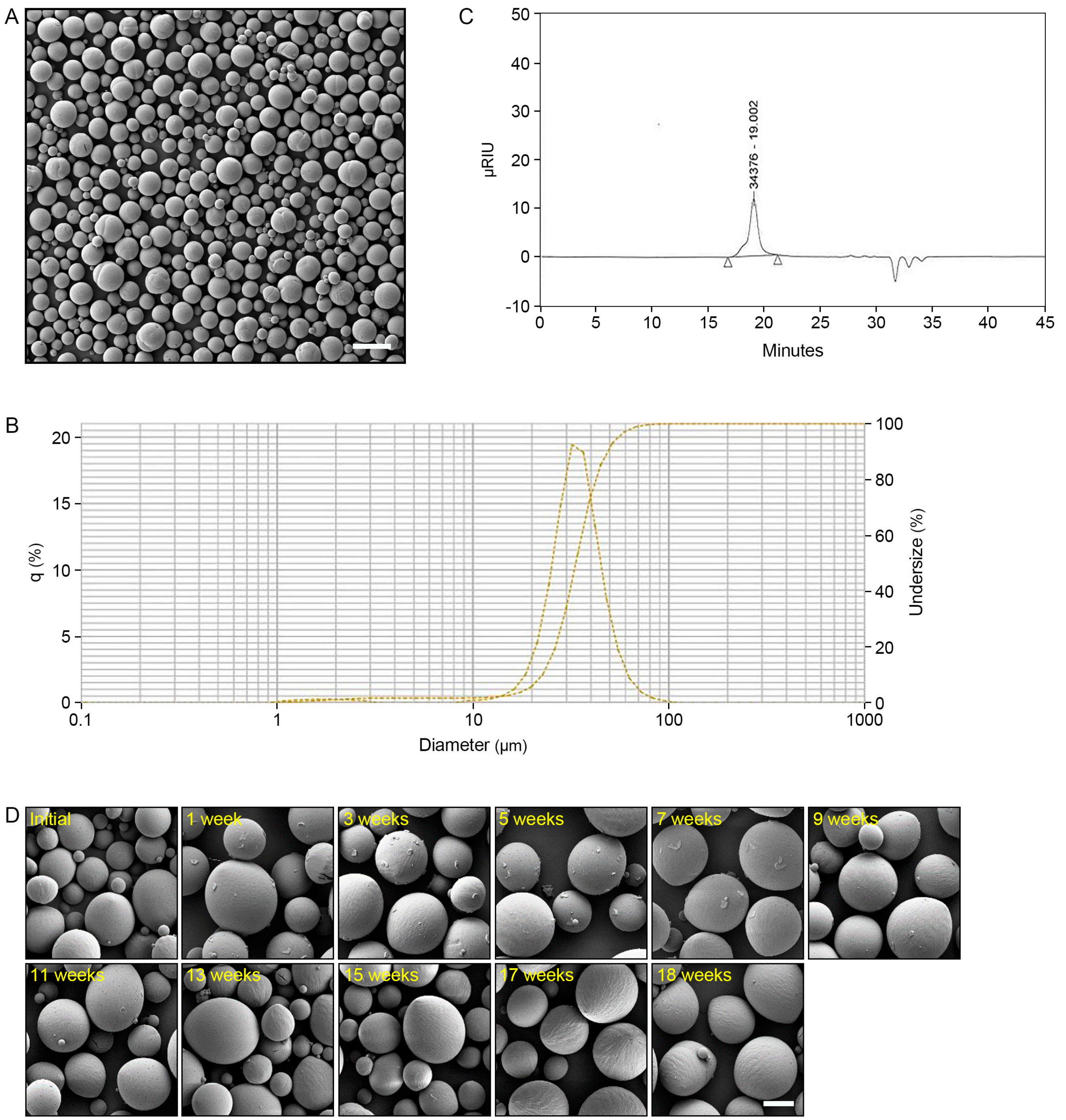

Figure S6. Characterization of PLLA microparticles. (A) Representative scanning electron microscopy (SEM) image showing spherical particle morphology. (B) Representative laser-scattering particle-size distribution, with a median particle diameter (D50) of 33.5198 µm. (C) Gel permeation chromatogram. The reported number-average molecular weight (Mn), weight-average molecular weight (Mw), and peak molecular weight (Mp) were 34,589, 38,945, and 34,376, respectively (calculated Mw/Mn = 1.126). (D) SEM images obtained at the initial time point and after 1, 3, 5, 7, 9, 11, 13, 15, 17, and 18 weeks of storage at 50 °C. The particles retained their spherical morphology without visible rupture or collapse under the tested conditions. This storage assessment evaluated visible particle morphology only. PLLA, poly-L-lactic acid.

Supplementary Tables

Table S1. List of antibodies used in this study.

Antibody	Company	Dilution rate		
		ELISA	Western blot	IHC
β-actin	Cell Signaling Technology	-	1:1,000	-
VEGF	Fine Test	-	1:1,000	-
VEGFR2	Cell Signaling Technology	-	1:1,000	-
NSD2	GeneTex	-	1:1,000	-
KDM2A	Abcam	-	1:1,000	-
H3K36me2	GeneTex	-	1:1,000	-
TGF- β1	Stressmarq	-	1:1,000	-
pSMAD2/3	Cell Signaling Technology	-	1:1,000	-
SMAD2/3	Cell Signaling Technology	-	1:1,000	-
α-SMA	Invitrogen	-	1:1,000	1:100
COL1	Santa Cruz Biotechnology	1:500	-	1:100
COL3	Santa Cruz Biotechnology	1:500	-	1:100

Table S2. List of primers for quantitative real-time PCR.

**Gene *(Organism)***		**Primer sequences**
ACTB (human)	Forward	5'- GGA CCT GAC TGA CTA CCT CAT G -3′
	Reverse	5'- AGC TTC TCC TTA ATG TCA CGC A -3′
NSD2 (human)	Forward	5'-TTC TGT CAA AAA CAC AGG GAT G-3′
	Reverse	5'-ACC TGA GCA GCT CTT CAA TCT C-3′
KDM2A (human)	Forward	5'-CTG CAC CTT ACA AAC AGC AGT C-3′
	Reverse	5'-GCA TTT CCT CAA AGT TTC AAC C-3′
TGFB1 (human)	Forward	5'-CCC ACA ACG AAA TCT ATG ACA A-3′
	Reverse	5'-GGA GCT CTG ATG TGT TGA AGA A-3′
SNAIL (human)	Forward	5'-TCA GAT GAG GAC AGT GGG AAA G-3′
	Reverse	5'-AGT AGA GGA GAA GGA CGA AGG A-3′
SLUG (human)	Forward	5'-TGC GAT GCC CAG TCT AGA AAA T-3′
	Reverse	5'-ATG TGT GGG TCC GAA TAT GCA T-3′
TWIST (human)	Forward	5'-GTC TTA CGA GGA GCT GCA GAC-3′
	Reverse	5'-CCA GCT TGA GGG TCT GAA TCT T-3′
Actb (mouse)	Forward	5'- CCT CAT GAA GAT CCT GAC CGA G -3′
	Reverse	5'- TTT GAT GTC ACG CAC GAT TTC C -3′
Snail (mouse)	Forward	5'-TGT GAA GAG ATA CCA GTG CCA G-3′
	Reverse	5'-AGA CTC TTG GTG CTT GTG GAG-3′
Slug (mouse)	Forward	5'-CCA AGG ATC ACA GTG GTT CAG A-3′
	Reverse	5'-GAA ACT TCT CAG CTT CGA TGG C-3′
Twist (mouse)	Forward	5'-CAA GCG CGG CAA GAA ATC TG-3′
	Reverse	5'-GCC AGT TTG AGG GTC TGA ATC T-3′

Supplementary Methods

Characterization procedures and data were provided by the manufacturer. McCoom7 PLLA microparticles (SACCI Bio Co., Ltd.) were sterilized by electron-beam irradiation before use.

For scanning electron microscopy (SEM), microparticle powder was mounted on conductive copper tape and coated with platinum under an argon atmosphere. Images were acquired at room temperature using a scanning electron microscope (Carl Zeiss, Jena, Germany) at an accelerating voltage of 15 kV (Figure S5A).

Particle-size distribution was analyzed using a laser-scattering particle size analyzer (LA-350; HORIBA, Kyoto, Japan). Microparticle powder was dispersed in water and analyzed according to the manufacturer's instructions (Figure S5B).

Molecular weight distribution was determined by gel permeation chromatography using a Waters Arc HPLC system equipped with a refractive index detector (Waters Corporation, Milford, MA, USA). PLLA was dissolved in tetrahydrofuran (THF) at 1% (w/v) and filtered through a 0.2 μm syringe filter. Styragel HR 1 THF, HR 3 THF, and HR 4 THF columns were connected in series and maintained at 40 °C. THF was used as the mobile phase under isocratic conditions at a flow rate of 0.5 mL/min. The detector cell temperature was maintained at 40 °C. The injection volume was 20 μL, and the total run time was 60 min (Figure S5C).

Morphological stability was assessed by SEM during storage at 50 °C at the initial time point and after 1, 3, 5, 7, 9, 11, 13, 15, 17, and 18 weeks. This assessment evaluated visible particle morphology only (Figure S5D).

The manufacturer reported a direct-contact cytotoxicity evaluation using L-929 mouse fibroblasts according to ISO 10993-5. Cellular lysis and morphological changes were assessed by visual inspection after 48 h of incubation, and cytotoxicity was graded according to the ISO 10993-5 criteria.

The manufacturer-provided documentation did not include crystallinity or quantitative dispersibility data.
